# Crovalimab: A Novel Approach in the Management of Paroxysmal Nocturnal Hemoglobinuria

**DOI:** 10.1002/hsr2.70986

**Published:** 2025-07-13

**Authors:** Laiba Jalal, Marium Ahmed, Anum Khalid

**Affiliations:** ^1^ Dow University Of Health Sciences Karachi Pakistan

**Keywords:** crovalimab, drug‐target‐drug complexes (DTDCs), FDA approval, paroxysmal nocturnal hemoglobinuria (PNH), SMART antibody

## Abstract

**Background and Aims:**

Paroxysmal Nocturnal Hemoglobinuria (PNH) is a rare acquired clonal blood disorder caused by mutations in the PIGA gene, leading to complement‐mediated hemolysis. Currently available terminal complement inhibitors, such as Eculizumab and Ravulizumab, pose several challenges, including the need for frequent intravenous infusions and the potential for resistance due to C5 polymorphisms. This study highlights the clinical significance of Crovalimab, a novel C5 inhibitor developed using SMART‐antibody technology, as a promising alternative.

**Methods:**

An extensive literature review was conducted using PubMed to evaluate the pharmacological properties, mechanism of action, and clinical trial data of Crovalimab. Phase 3 trials—COMMODORE 1, 2, and 3—were analyzed to assess Crovalimab's safety, efficacy, and potential benefits in both C5‐inhibitor naïve and previously treated patients.

**Results:**

Crovalimab demonstrated high bioavailability, an extended half‐life, and subcutaneous administration every 4 weeks, offering a better alternative to intravenous therapies. Unlike existing treatments, Crovalimab targets the C5 β‐chain, making it effective even in patients with the R885H polymorphism. The COMMODORE trials reported favorable outcomes, including effective hemolysis control, reduced transfusion dependence, and a manageable safety profile. Adverse events were mostly mild, with rare occurrences of transient immune complex reactions.

**Conclusion:**

Crovalimab represents a significant advancement in the management of PNH, with the potential to reduce treatment burden while maintaining efficacy. However, further research is required to evaluate its long‐term safety and effectiveness across diverse populations.

## 1 Introduction

Paroxysmal Nocturnal Hemoglobinuria (PNH) is a rare acquired clonal blood disease caused by a deficiency of phosphatidylinositol glycan class A (PIG‐A) protein, crucial for synthesizing a glycolipid known as glycosylphosphatidylinositol (GPI) anchor that attaches surface proteins to the cell membrane. In hematopoietic stem cells, mutations in the PIGA gene lead to the formation of daughter cells lacking GPI‐anchored proteins like CD55 and CD59 [1]. The affected cells become susceptible to lysis by the complement system, causing anemia and intravascular hemolysis. Disease complications include thromboembolic events, impaired renal function, abdominal pain, and pulmonary arterial hypertension [2]. PNH is estimated to affect 15.9 individuals per million globally, and diagnostic flow cytometry is considered the gold standard test for its diagnosis [3].

## 2 Current Therapeutic Options

Currently, bone marrow transplant is the sole PNH curative therapy, but it is not used as the first‐line treatment due to its morbidity and mortality. The primary treatment of symptomatic PNH includes proximal and terminal complement inhibitors. Proximal complement inhibitors, such as Pegcetacoplan, prevent the activation of C3 complement protein, while terminal complement inhibitors, such as Eculizumab and Ravulizumab, target C5 activation and are considered the current treatment options for symptomatic PNH in many countries. Despite their efficacy, these drugs place a significant burden on patients, as Eculizumab requires intravenous (IV) infusions every 2 weeks, often in combination with blood transfusions due to underlying bone marrow failure, as well as the risk of extravascular and breakthrough hemolysis. Many individuals continued to exhibit hemolysis while receiving Eculizumab, possibly due to incomplete C5 blockade resulting from underdosing. The introduction of Ravulizumab, a terminal inhibitor with an extended half‐life, addressed some of these issues, however, it was insufficient as in some patients, polymorphisms like c.2654 G → A (Arg885His) can stop the terminal complement inhibitors from binding to C5 [1]. Iptacopan, the first oral monotherapy for PNH, and Danicopan, an oral add‐on therapy to Eculizumab or Ravulizumab, are proximal complement inhibitors recently approved by the FDA. Iptacopan binds to factor B, C3, and C5 convertases of the Alternative pathway and prevents their activation. Danicopan binds reversibly to factor D to prevent its activation [4]. However, Iptacopan carries a black box warning for the risk of life‐threatening infections caused by encapsulated organisms [5].

## 3 Emergence of Crovalimab

To address these concerns, the Food and Drug Administration (FDA) has recently authorized Crovalimab, a C5 inhibitor antibody developed using the sequential monoclonal antibody recycling technology (SMART‐antibody), for PNH treatment [6]. It binds and inhibits the breakdown of C5 into C5a and C5b, thus preventing MAC formation [7]. Crovalimab is a drug designed using isoelectric point, neonatal Fc receptor, and pH‐dependent affinity engineering, and it binds to C5 effectively. It enhances endothelial cell uptake of C5‐bound Crovalimab and C5 disposal in the endosome. Additionally, this drug supports efficient recycling via neonatal Fc receptor‐mediated pathways [6]. Unlike Eculizumab and Ravulizumab, which target the alpha chain of C5, Crovalimab specifically binds to an antigenic determinant on the C5 β chain, a crucial component in the complement system and halts C5 convertase from cleaving C5. This feature benefits patients who exhibit poor responses to Eculizumab and Ravulizumab due to C5 R885H polymorphism. Crovalimab has a long half‐life, high bioavailability as well as high solubility, thus requiring small‐volume subcutaneous injections once in 4 weeks [2, 6]. Multiple trials have been conducted to evaluate Crovalimab, and the results have established its use in two major indications:

- C5‐inhibitor Naïve PNH patients: These are patients who have received no prior treatment with C5 inhibitors. The COMMODORE 2 and 3 trials evaluated Crovalimab's safety and efficacy in this population.
- PNH patients switching from other C5 inhibitors: These are patients who were being treated with C5 inhibitors such as Eculizumab and were then switched to Crovalimab. The COMMODORE 1 trial assessed Crovalimab's safety profile in this group of patients.

### 3.1 The Details of These Trials Are Mentioned Below

The Phase 3 COMMODORE 1 study evaluated the safety, pharmacodynamics, pharmacokinetics, and exploratory efficacy of Crovalimab versus Eculizumab in PNH patients receiving C5 inhibitors. It randomized 89 patients to 2 groups: 45 patients received Crovalimab while 44 continued with Eculizumab. Adverse events were reported in 77% and 68% of the patients treated with Crovalimab and Eculizumab, respectively. The most common ones were pyrexia (16% with Crovalimab vs. 2% with Eculizumab), COVID‐19 (14% vs. 17%), and infusion‐related reactions (14% vs. 0%), respectively. The most common symptom of infusion‐related reactions was headache (5%) [2]. AEs that occurred exclusively in the Crovalimab arm due to the switch from Eculizumab or subcutaneous administration included injection site reactions, most common of which were rash, arthralgia and/or myalgia, with no evidence of renal involvement and transient immune complex reactions (also known as drug‐target‐drug‐complexes (DTDCs)) [2]. DTDCs may form when residual Eculizumab binds to C5, interfering with Crovalimab's binding and clearance. This can lead to delayed therapeutic effects and the occurrence of transient immune reactions as well. The clearance of these DTDCs from the serum depends on their sizes. Modeling has shown that smaller DTDCs are eliminated much more quickly than larger DTDCs. The shifting size of DTDCs was explored by keeping the lattice theory of immune complex formation in mind. An initial loading dose, followed by once‐weekly, four subcutaneous loading doses, and then a maintenance subcutaneous dose every 4 weeks, was given. Simulation outcomes predicted a reduced serum concentration of large DTDCs [7]. Only one patient in COMMODORE 1 had a severe transient immune complex reaction, which included symptoms of arthralgia, dizziness, abdominal pain, and nausea. Treatments used for mild/moderate reactions were mainly analgesics or nonsteroidal anti‐inflammatories, antihistamines, and topical and systemic steroids for symptom relief [2]. The occurrence of DTDCs in C5 inhibitor‐experienced patients, as highlighted by Nishimura et al., warrants careful monitoring when transitioning therapies [7].

COMMODORE 2 was a global, randomized, open‐label, multi‐center phase 3 trial that compared Crovalimab and Eculizumab in PNH patients naive to complement inhibition. The results showed that Crovalimab was non‐inferior to Eculizumab for both co‐primary endpoints of hemolysis control and transfusion avoidance. The mean proportion of patients with hemolysis control was 79.3% with Crovalimab and 79.0% with Eculizumab. Crovalimab was also non‐inferior to Eculizumab for the secondary endpoints of breakthrough hemolysis and hemoglobin stabilization. The proportion of patients with hemoglobin stabilization was 63.4% (85 of 134 patients) in the Crovalimab arm and 60.9% (42 of 69 patients) in the Eculizumab arm, thereby, confirming non‐inferiority of Crovalimab to Eculizumab for this endpoint [6].

A Phase 3 single‐arm COMMODORE 3 study was conducted to evaluate the safety and efficacy of Crovalimab. In this study, 51 patients suffering from PNH, who received no previous complement inhibitor therapy, were given a loading dose of Crovalimab on Day 1, comprising one intra‐venous infusion (weight < 100 kg: 1000 mg; weight ≥ 100 kg: 1500 mg), followed by a series of four 340 mg subcutaneous injections (once weekly, starting on Day 2 through Week 4). Maintenance dosing was started at Week 5, comprising subcutaneous injections (weight < 100 kg: 680 mg; weight ≥ 100 kg: 1020 mg) given every 4 weeks. Crovalimab proved to be an efficacious treatment option for patients with PNH since it was able to achieve a transfusion‐sparing rate as compared to the previous observations in PNH‐affected individuals with no prior treatment (51%; 22 of 43 patients) [1]. Similarly, COMMODORE 1 and COMMODORE 2 studies also reported a favourable benefit‐risk profile of Crovalimab. The adverse effects noted in participants included mild site reactions. Only one serious adverse effect of bacteremia was reported in one participant of the COMMODORE 3 study [1, 2].

## 4 Conclusion

Across all three trials, Crovalimab has shown promising results, particularly its ability to reduce hemolysis and significantly lower transfusion rates, all while requiring fewer administrations. The mild adverse effect profile of Crovalimab further enhances its potential to revolutionize PNH treatment and diminish the burden on patients.

However, further research is needed to evaluate the efficacy of Crovalimab across different age groups, ethnicities, and genders. This will ensure its broad applicability and maximize its potential benefits for diverse patient populations.

## Author's Perspective

The introduction of Crovalimab marks a significant step forward in expanding therapeutic options for patients with PNH, particularly those who face challenges with existing C5 inhibitors. Its subcutaneous administration and extended dosing interval offer substantial benefits in reducing treatment burden and improving patient quality of life. Crovalimab's favorable adverse effect profile, characterized primarily by mild injection site reactions and manageable immune complex‐related events observed especially during switching from other C5 inhibitors, further supports its clinical utility. Moreover, its unique binding to the C5 β chain provides an effective alternative for patients with C5 polymorphisms that compromise response to other therapies. Further research in diverse patient populations will be critical to fully establish Crovalimab's long‐term safety and efficacy and to guide its optimal clinical use.

## Author Contributions

**Laiba Jalal:** conceptualization, writing – original draft, validation, supervision, formal analysis. **Marium Ahmed:** writing – original draft, formal analysis, validation. **Anum Khalid:** writing – review and editing, validation, formal analysis.

## Ethics Statement

The authors have nothing to report.

## Conflicts of Interest

The authors declare no conflicts of interest.

## Transparency Statement

The lead author Laiba Jalal affirms that this manuscript is an honest, accurate, and transparent account of the study being reported; that no important aspects of the study have been omitted; and that any discrepancies from the study as planned (and, if relevant, registered) have been explained.

## Data Availability

The authors have nothing to report.
